# Supplementary material for: Drug Metabolism of Hepatocyte-like Organoids and Their Applicability in In Vitro Toxicity Testing
Source: Molecules. 2023 Jan 7;28(2):621. doi: 10.3390/molecules28020621 (PMC9867526; doi:10.3390/molecules28020621)
Supplement: Supplementary file 1 [file molecules-28-00621-s001.zip › Supplemental data_Bouwmeester.pdf]

Supplemental data

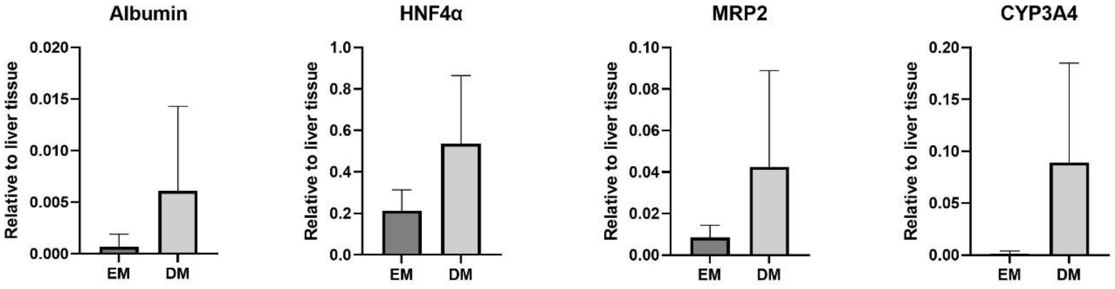

Figure S1. Gene expression of hepatic markers upon hepatic differentiation of ICOs. Gene expression levels are shown relative to levels in human liver tissue. EM: ICOs in expansion condition. DM: ICOs differentiated towards HL-ICOs. HNF4α, Hepatocyte Nucleus Factor 4 alpha; MRP2, Multi Resistance Protein 2; CYP3A4, Cytochrome P450 3A4. Expression is shown for multiple donors (n=6). Error bars represent the standard deviation.

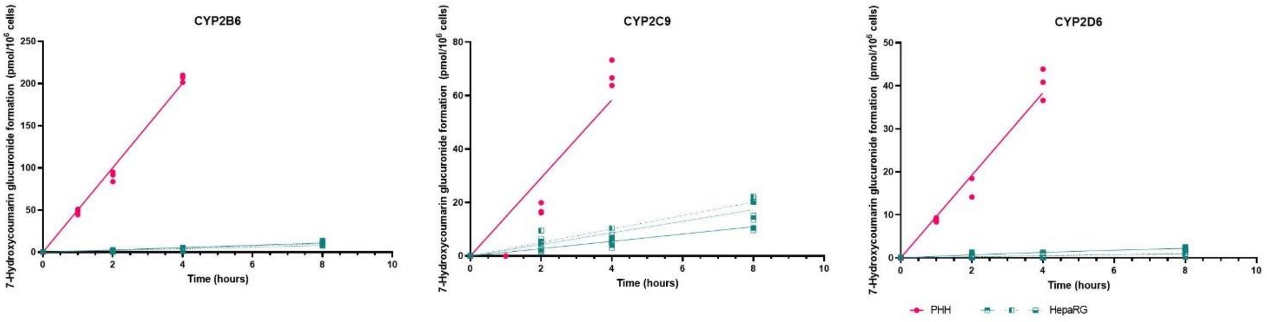

Figure S2. Metabolite formation of bupropion (CYP2B6), tolbutamide (CYP2C9), and dextrometrorphan (CYP2D6) in primary human hepatocytes and HepaRGs. HepaRG: Each green symbol represents an independent experiment. PHH: Technical triplicates are shown in pink.
